# Freckles

**Published:** 1885-12-20

**Authors:** 


**Freckles.**—(Medical World) Freckles are said to yield to equal parts of lactic acid and glycerine, frequently applied. Probably it is effectual, as the same mixture dissolves diphtheritic false membranes. They may also be removed by touching them with a cystal of carbolic acid when dry.

